# Experimental data of structural tests on non-continuously stiffened steel panels subjected to uniform compression

**DOI:** 10.1016/j.dib.2025.111644

**Published:** 2025-05-14

**Authors:** Immo Lukas, Ralph Timmers, Melanie Ropele, Robert Lang

**Affiliations:** Faculty of Engineering Sciences, Unit of Steel Construction and Mixed Building Technologies, University of Innsbruck, Technikerstrasse 13, Innsbruck 6020, Austria

**Keywords:** Plate buckling, Imperfections, DIC-measurement, Triangular-shaped buckling mode, Load-bearing behavior

## Abstract

This data article presents experimental datasets from structural tests of 20 open-section and 30 closed-section non-continuously stiffened steel panels subjected to uniform compression. Non-continuous longitudinal stiffening denotes a construction method of flat plates in which the plate’s stiffeners end within the panel, i.e., before reaching the transverse stiffener or the end of the girder. The related research article ‘Experimental investigation on stiffened plates with non-continuous longitudinal stiffeners ending within the panel’ provides a detailed description of the testing program. The dataset includes the measured load-displacement curves, tensile coupon tests, and measured initial geometric imperfections. Therefore, providing all necessary data to both validate a numerical model and steer future experimental work for other load cases. Moreover, a one-page overview yields all relevant data for each of the 50 tested specimens.

Specifications Table

**Table:** 

Subject	Engineering & Materials science
Specific subject area	Structural experimental testing
Type of data	Tables, Graphs/Figures, CSV-files (test results), XLSX-files (tensile coupon tests), STL-files (imperfections)
Data collection	The experimental tests were conducted on a Hydropuls Schenck Instron POZ 851, model PL 1.6 K, with a GTM series RF load cell (accuracy class 0,05). All tests were performed displacement controlled with a constant displacement rate of 0,01mm/s. The axial load is measured by the Hydropuls load cell. Four vertical resp. one horizontal linear variable displacement transducer (LVDTs) are used to measure in-plane resp. out-of-plane displacements. On the two twice-designed specimens (*V_n1_g20_lsw2_open* and *V_n3_g05_lss2_open*), strain gauges (SGs) were installed. The SGs data serves as a control measurement because the plastic strain position cannot be predicted a priori. Digital image correlation (DIC) was used to record the displacement field before and during the tests. The ARAMIS HS Rev.02 (1280×1024 pixels) by GOM possesses a pair of digital cameras with Schneider Kreuznach Titanar B24 lenses (focal length 24 mm). The calibration protocol attested a stereo-angle of 25° with a camera distance of 688 mm and a specimen-camera distance of 2000 mm. The calibration deviation reached 0,025 pixels (permitted limit: 0,050 pixels).
Data source location	Institution: University of Innsbruck City/Town/Region: Innsbruck Country: Austria
Data accessibility	Repository name: Research Data UIBK Data identification number: 10.48323/vkc96-nrz42 Direct URL to data: https://researchdata.uibk.ac.at//records/vkc96-nrz42
Related research article	I. Lukas, R. Timmers, M. Ropele, R. Lang, Experimental investigation on stiffened plates with non-continuous longitudinal stiffeners ending within the panel, Thin-Walled Structures 193 (2023) 111260 https://doi.org/10.1016/j.tws.2023.111260

## 1 Value of the Data

• The data presents the first-ever conducted experimental campaign for non-continuously stiffened steel panels [1], including an overview page for each test specimen.
• The experimental data provide all required information (tensile coupon tests, imperfections, load-displacement curves) for numerical calibration and validation purposes using geometrically and materially non-linear analysis with imperfections (GMNIA).
• The data may build the baseline for further experimental investigations in terms of different geometries or load cases.
• The experimental data can be taken to assess and interpret one’s own simulations and compared to results from [2].
• As the contribution examines a new construction method to extend current design guidelines (such as EN 1993-1-5), the data helps to elaborate further problem solving of this matter.

## 2 Background

In bridge construction, longitudinal stiffeners of plated girders are typically designed to either extend continuously along the entire girder or to span discontinuously between transverse stiffeners. This research focuses on a design approach commonly used in crane manufacturing – non-continuous stiffening. In non-continuous longitudinal stiffening, the stiffeners end within the panel, stopping before reaching the transverse stiffener or girder end. This construction technique is beneficial in terms of efficient material usage and more straightforward fabrication, as it eliminates the need for complex intersections, cut-outs, and butt welding of the longitudinal stiffeners. Simultaneously, non-continuous stiffening features certain drawbacks. The eccentricity between the load application point on the plate and the center of gravity of the entire panel introduces an additional bending moment. The abrupt change in stiffness at the stiffener’s end results in localized stress concentrations. Current standardization of EN 1993-1-5 [3] is limited to conventional, continuous longitudinal stiffening. This experimental data lays the foundation for a normative extension. Similarly, there are several reasons why this construction method is gaining popularity, particularly in mechanical engineering. The primary factor driving its adoption is the advantages it offers in the manufacturing process. One key benefit of this design is that it allows for easy, continuous welding around the longitudinal stiffeners. Additionally, there is no need for intricate transverse stiffeners or web intersections, which typically require manual adjustments and often lead to challenges in maintaining permissible tolerances. Moreover, the connection between the stiffeners and the transverse stiffener must be manually welded from both sides. The most fatigue-sensitive areas, in particular, are rarely welded smoothly, as achieving a continuous weld around these points is difficult. Manufacturers are often forced to interrupt the process and restart from the opposite side, which creates additional notches. Furthermore, when using non-continuous stiffeners, sealing welds for the structure become significantly easier. In addition, the individual sections of a bridge or crane must be welded together on-site, which becomes significantly easier when utilizing non-continuous stiffeners in terms weldability.

Another contributing factor is the significant gap in research literature regarding non-continuous stiffening. The studies conducted by de Pauw et al. [[4], [5], [6]] focus solely on linear-elastic analyses without evaluating the actual load-bearing behavior or structural capacity. Conversely, Unterweger et al. [7,8] perform numerical ultimate load simulations incorporating imperfections (GMNIA) on structural components with terminating longitudinal stiffeners. Their research examines the impact of longitudinal stiffeners that extend discontinuously from one transverse stiffener to the next. However, they do not specifically address the case of non-continuous longitudinal stiffening as considered here. The PhD Thesis by [9] explores the reinforcement of flat steel panels using adhesively bonded stiffeners, which are typically designed as non-continuous. However, the primary emphasis of this study is on analyzing the bonded joint. While the effect of non-continuous stiffeners on the panel’s buckling behavior is acknowledged, it is not examined in depth. Instead, constructive solutions to mitigate the issue—such as additional bonding elements or angles—are proposed.

## 3 Data Description

**Fig. 1:**
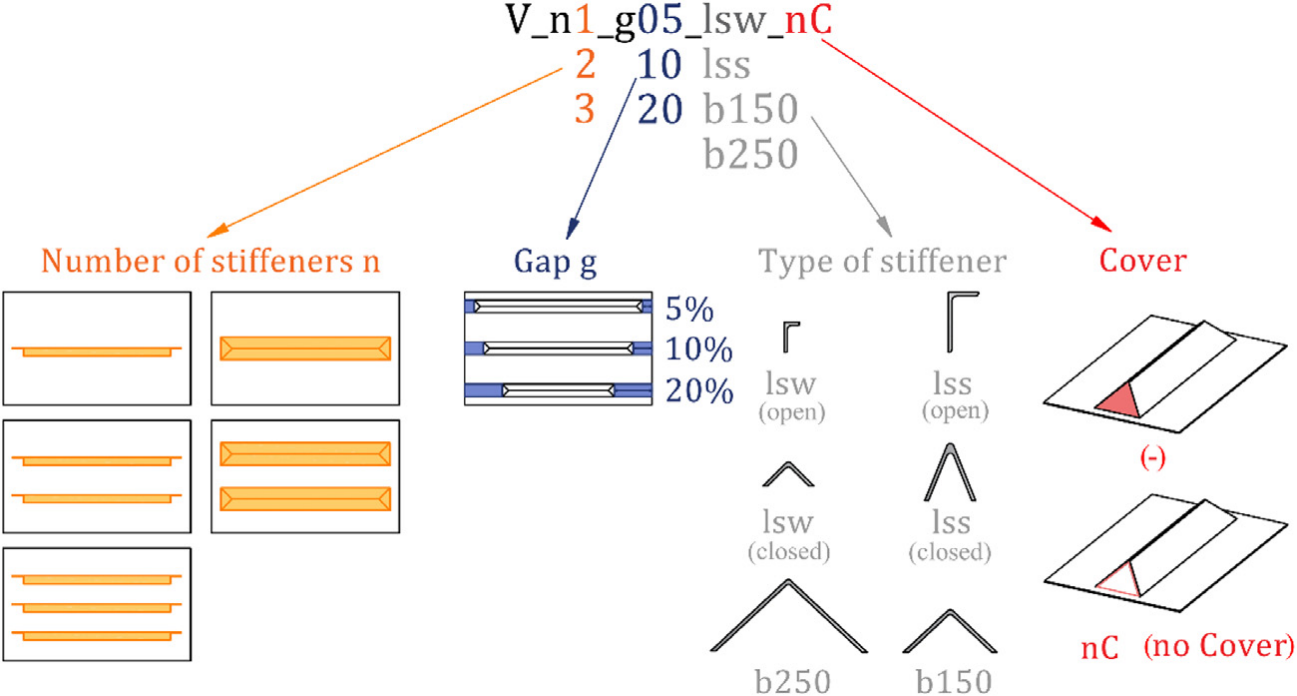
Overview of the geometry parameters [1].

**Fig. 2:**
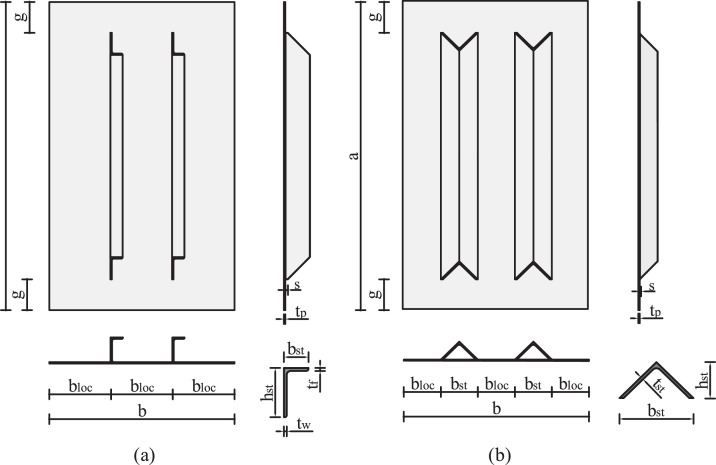
Definition of the test specimens, exemplarily for n=2 stiffeners: (a) Open-section stiffener types, (b) closed-section stiffener types [1].

Fig. 1 gives an overview of all geometry parameters considered and details the specimen naming. Information about all constructed specimens is presented in Table 1, in which *n* denotes the number of stiffeners, the gap is defined as $gap=\frac{g}{a}$ (as visualized in Fig. 2), the relative stiffness of the stiffener $\gamma_{sl,1}$, and the section type. In the case of closed sections, a cover (*t=3mm*) was considered for several specimens for comparative reasons. The relative stiffness of a stiffener is defined as $\gamma_{sl,1}=\frac{I_{sl,1}}{I_{p}}$, with the moment of inertia of a single stiffener, including the contributing plate part *I_sl,1_* and the moment of inertia for plate bending $I_{p}=\frac{bt^{3}}{12(1-\nu^{2})}$.

**Table 1:** Geometry information of the specimens [1].

Specimen (-)	n (-)	gap (%)	γ_sl,1_ (-)	section type (-)
V_n1_g05_lss	1	5	200 (lss)	open / closed / closed nC
V_n1_g05_lsw	1	5	25 (lsw)	open / closed / closed nC
V_n1_g05_b250	1	5	400 (b250)	closed / closed nC
V_n2_g05_lss	2	5	200 (lss)	open / closed / closed nC
V_n2_g05_lsw	2	5	25(lsw)	open / closed / closed nC
V_n2_g05_b150	2	5	100 (b150)	closed / closed nC
V_n3_g05_lss	3	5	200 (lss)	open (2x)
V_n3_g05_lsw	3	5	25 (lsw)	open
V_n1_g10_lss	1	10	200 (lss)	open / closed
V_n1_g10_lsw	1	10	25 (lsw)	open / closed
V_n1_g10_b250	1	10	400 (b250)	closed
V_n2_g10_lss	2	10	200 (lss)	open / closed
V_n2_g10_lsw	2	10	25 (lsw)	open / closed
V_n2_g10_b150	2	10	100 (b150)	closed
V_n3_g10_lss	3	10	200 (lss)	open
V_n3_g10_lsw	3	10	25 (lsw)	open
V_n1_g20_lss	1	20	200 (lss)	open / closed / closed nC
V_n1_g20_lsw	1	20	25 (lss)	open (2x) / closed / closed nC
V_n1_g20_b250	1	20	400 (b250)	closed / closed nC
V_n2_g20_lss	2	20	200 (lss)	open / closed / closed nC
V_n2_g20_lsw	2	20	25 (lsw)	open / closed / closed nC
V_n2_g20_b150	2	20	100 (b150)	closed / closed nC
V_n3_g20_lss	3	20	200 (lss)	open
V_n3_g20_lsw	3	20	25 (lsw)	open

The employed types of stiffeners (Fig. 1) result from an EN 1993-1-5 lower-limit recommendation of $\gamma_{sl,1}=25$ (lsw – **l**ongitudinal **s**tiffener **w**eak), and a freely chosen high value of $\gamma_{sl,1}=200$ (lss – **l**ongitudinal **s**tiffener **s**trong). The stiffener types b150 and b250 arise from arranging stiffened and unstiffened widths of the plate equally (*b_st_ = b_loc_*). All stiffeners are tapered at an angle of 45° to smoothen the change in stiffness and have a vertical stub (*s=10mm*) to facilitate weldability (Fig. 2).

Varying all stiffener characteristics, while keeping the plate dimensions constant, facilitates examining a broad range of specimens without the need to change the test setup. In real-world applications in crane manufacturing, mostly open sections are employed. Likewise, the use of closed sections is popular in bridge design due to adventurous properties in rotational stiffness.

All folders of the repository are subdivided into “open” and closed”, referring to open- resp. closed-section stiffeners. Details on the specimen naming are given in Fig. 1.

Folder **“01_overview_results_specimen”** contains a one-page overview (pdf-file) of all relevant experimental test data for each specimen (measured geometry, material data, ultimate load, load-displacement curves with out-of-plane deformations), as demonstrated in Fig. 10.

Folder **“02_tensile_coupon_test”** contains xlsx-files of the tensile coupon test with the columns:

• Time (s): time measurement of the coupon test
• Dist (mm): applied machine distance (displacement-controlled)
• Force (N): measured resulting force
• Length (mm): measured coupon deformation with extensometer
• Strain (%): resulting engineering strain
• Stress (N/mm²): resulting engineering stress
• Strain_cauchy (%): resulting true/Cauchy strain
• Stress_cauchy (N/mm²): resulting true/Cauchy stress

The subfolder “open” contains coupon tests for all open-section specimens for the plates, stiffener webs, and stiffener flanges, as no test certificates have been provided. The subfolder “closed” comprises test certificates (pdf-files), including the chemical composition of S235JR and 10 random control coupon tests for each plate and stiffener.

Folder **“03_geometric_imperfection_data”** contains STL-files, which are cleaned from outliers and centered to the coordinate system. Fig. 9 shows an example of an STL-file point cloud data applied to the corresponding finite element model for easier interpretation of the imperfection shape.

Folder **“04_test_result_data”** contains csv-files of the test results with the columns:

• Time (s): time measurement of the hydraulic piston
• Dist (mm): applied vertical distance (displacement-controlled)
• Force (N): measured resulting force
• Force_kN (kN): measured resulting force
• Dist_aramis (mm): DIC-measured vertical displacement from ARAMIS
• WA_1_top (mm): vertical displacement of V-1 (see Fig. 4)
• WA_2_top (mm): vertical displacement of V-2 (see Fig. 4)
• WA_3_bottom (mm): vertical displacement of V-3 (see Fig. 4)
• WA_1_bottom (mm): vertical displacement of V-4 (see Fig. 4)
• WA_5_horizontal (mm): horizontal displacement of H-5 (see Fig. 4)
• WA_vertical_mean (mm): mean value of V-1 and V-2

Specimens *V_n1_g20_lsw2_open* and *V_n3_g05_lss2_open* additionally contain:

• DMS 1-21 (µm/m): strain measured by strain gauge 1-21 (21 columns)
  ○ With 1-7: top row, left to right (Fig. 8)
  ○ With 8-14: middle row, left to right (Fig. 8)
  ○ With 15-21: bottom row, left to right (Fig. 8)

## 4 Experimental Design, Materials and Methods

### 4.1 Test setup and procedure

**Fig. 3:**
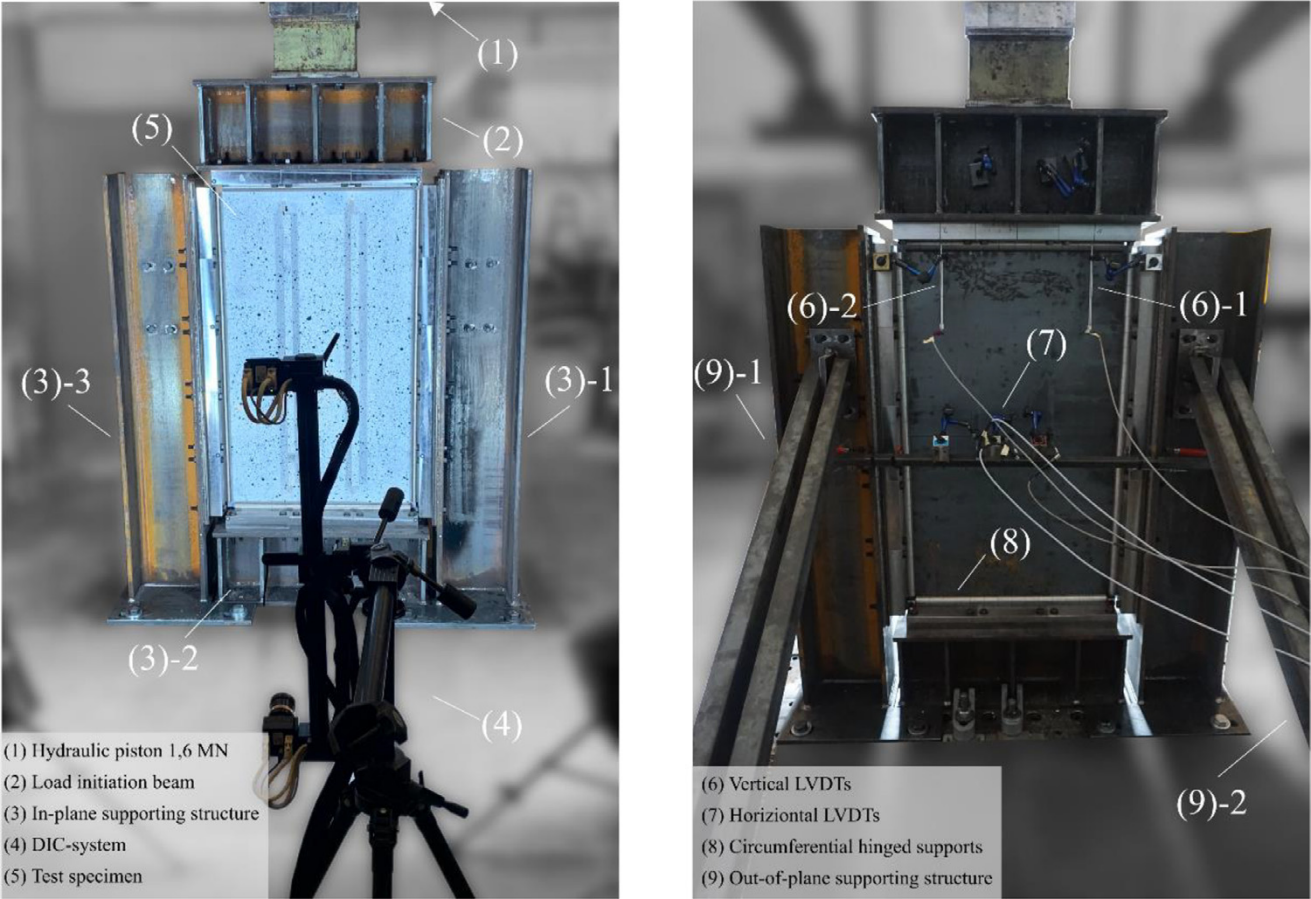
Overview of the laboratory test setup including instrumentation [1].

**Fig. 4:**
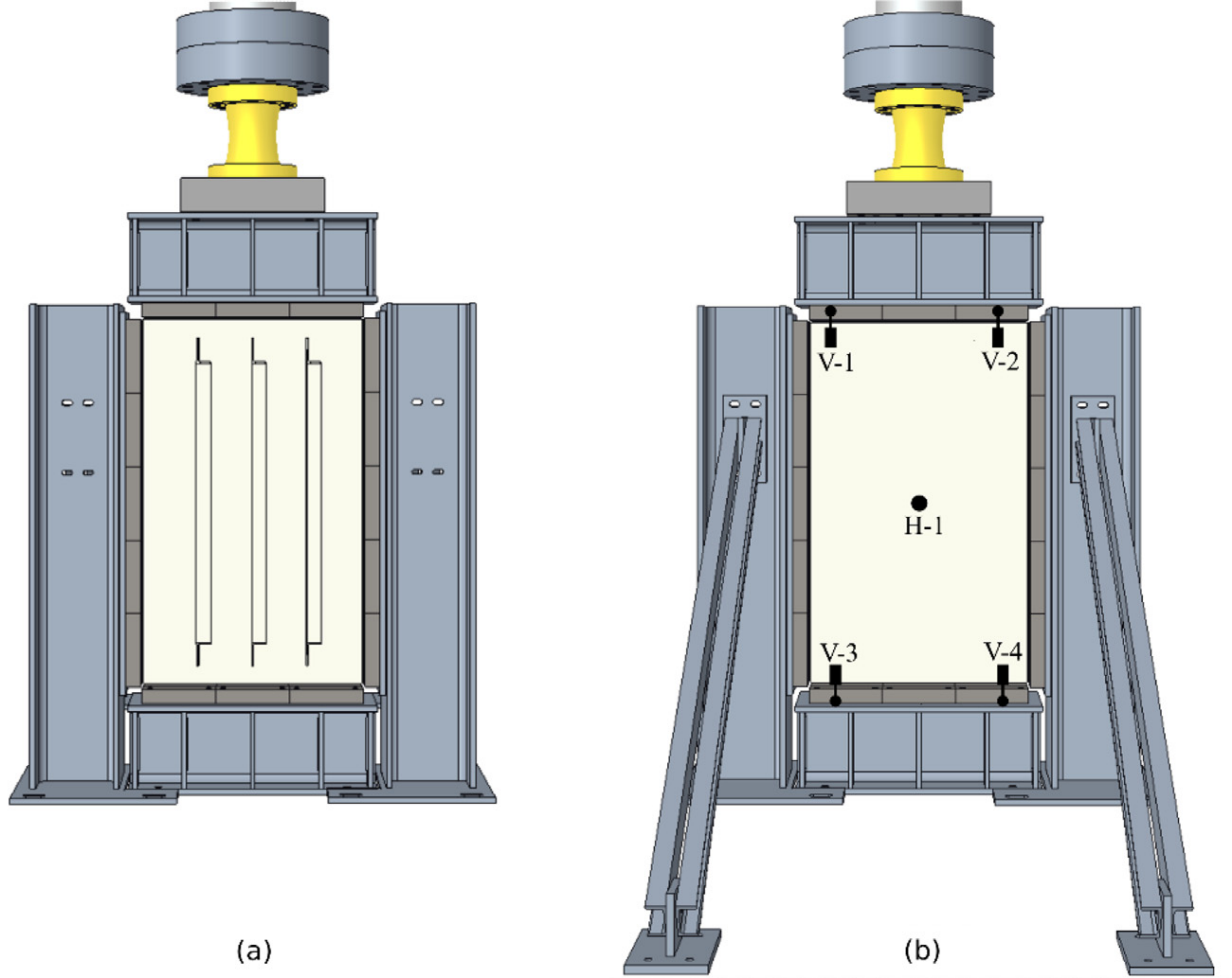
Digital model of the test layout including LVDTs positioning: (a) Frontside view, (b) backside view [1].

**Fig. 5:**
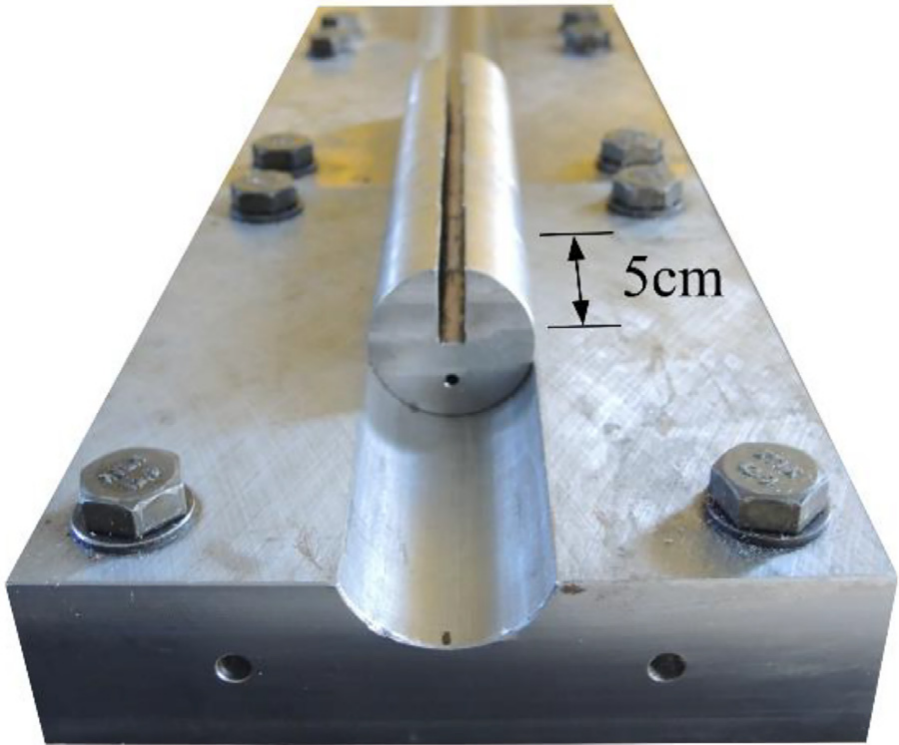
All around hinged support by a roller-bearing block (modified from [1]).

Fig. 3, Fig. 4 show the test layout for the panel compression test. The frame (Fig. 3 (3)) is made of S355 steel sections which are additionally stiffened. The lateral supporting structure (Fig. 3 (9)) prevents out-of-plane deformations. A roller-bearing block construction, as shown in Fig. 3 (8) and Fig. 5, supports the test specimens with hinged boundary conditions along all plate edges. For production reasons, it was not possible to manufacture the rollers in pieces smaller than 5 cm. Bridge-bearing grease has been applied to the rollers to decrease further friction.

The plate is loaded uniformly in-plane with the hydraulic piston (Fig. 3 (1) and (2)) Hydropuls Schenck Instron POZ 851, model PL 1.6 K, with a GTM series RF load cell of accuracy class 0,05. The specimens have been designed in preliminary studies [10] to comply with a maximum capacity of 1,6 MN. The load is applied displacement-controlled with an operating rate of 0,01 mm/s.

### 4.2 Instrumentation

The load cell of the hydraulic piston measures the applied force at a rate of 0,2s. Digital image correlation (DIC), Fig. 3 (4), monitors the panels’ deformation state. Four vertical and one horizontal linear variable displacement transducers (LVDTs) track the displacements, as shown in Fig. 4. The strongest and the weakest specimen configuration of the open-section test series have been produced twice (compare Table 1) and equipped with strain gauges according to Fig. 8, serving as control measurements.

**Fig. 6:**
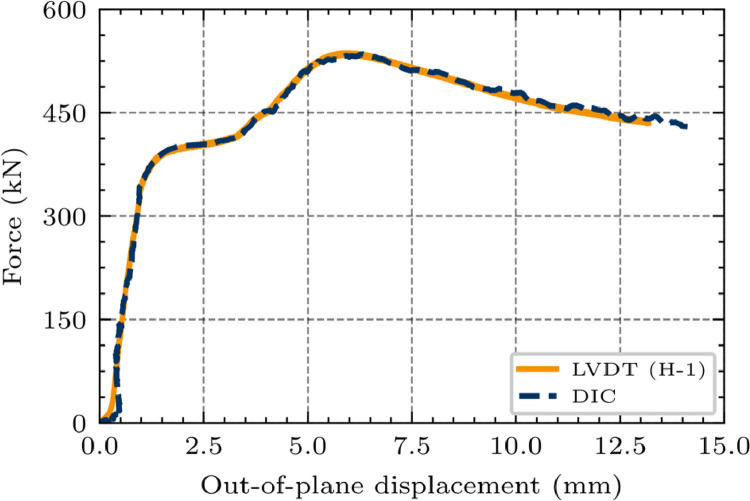
Validation of the DIC measurement: Comparison of LVDT (H-1) and DIC on the example of specimen V_n2_g20_lsw_open [1].

**Fig. 7:**
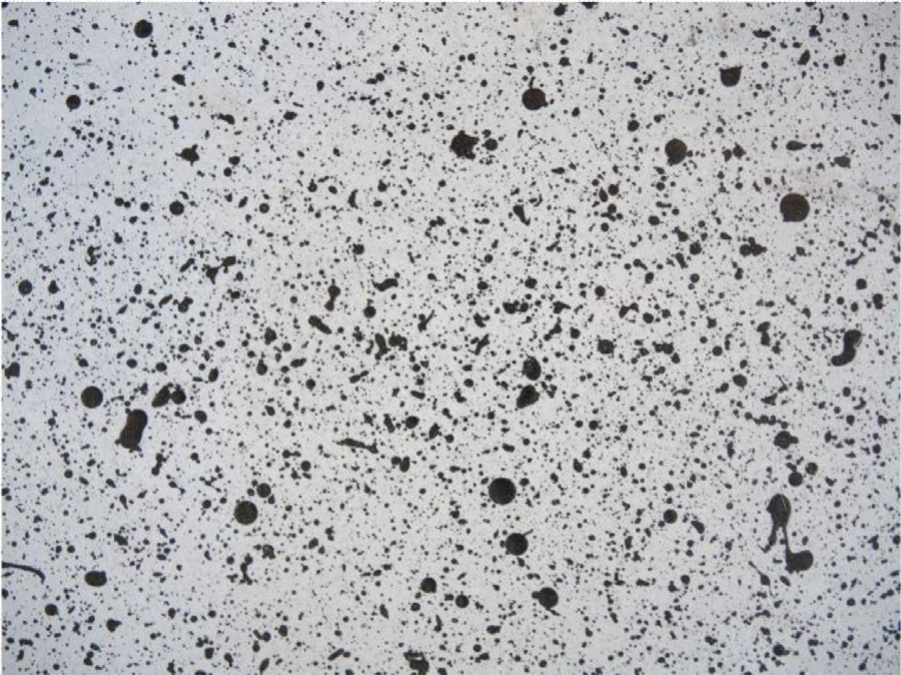
Random dot pattern to track the specimens’ displacement field with DIC.

The DIC-system ARAMIS HS Rev. 02 (1280×1024pixels) by GOM uses CCD cameras (charge-coupled devices), with the specification Kreuznach Titanar B24 (focal length 24mm) to continuously record the displacement field using triangular algorithms. This requires a random, non-repetitive dot pattern of different sizes applied to the surface of the test specimens (Fig. 7). The DIC-system demands a calibration protocol allowing a deviation of 0,05 pixels, resulting in a measuring tolerance of 0,009mm for a measuring volume of 1380 / 1160 / 1160 mm. Setting up a stereo-angel of 25° with a camera distance of 688mm and a specimen-to-camera distance of 2000mm results in a deviation of 0,025 pixels. Several DIC-results have been validated with vertical and horizontal LVDTs and strain gauge results, as exemplarily demonstrated in Fig. 6.

**Fig. 8:**
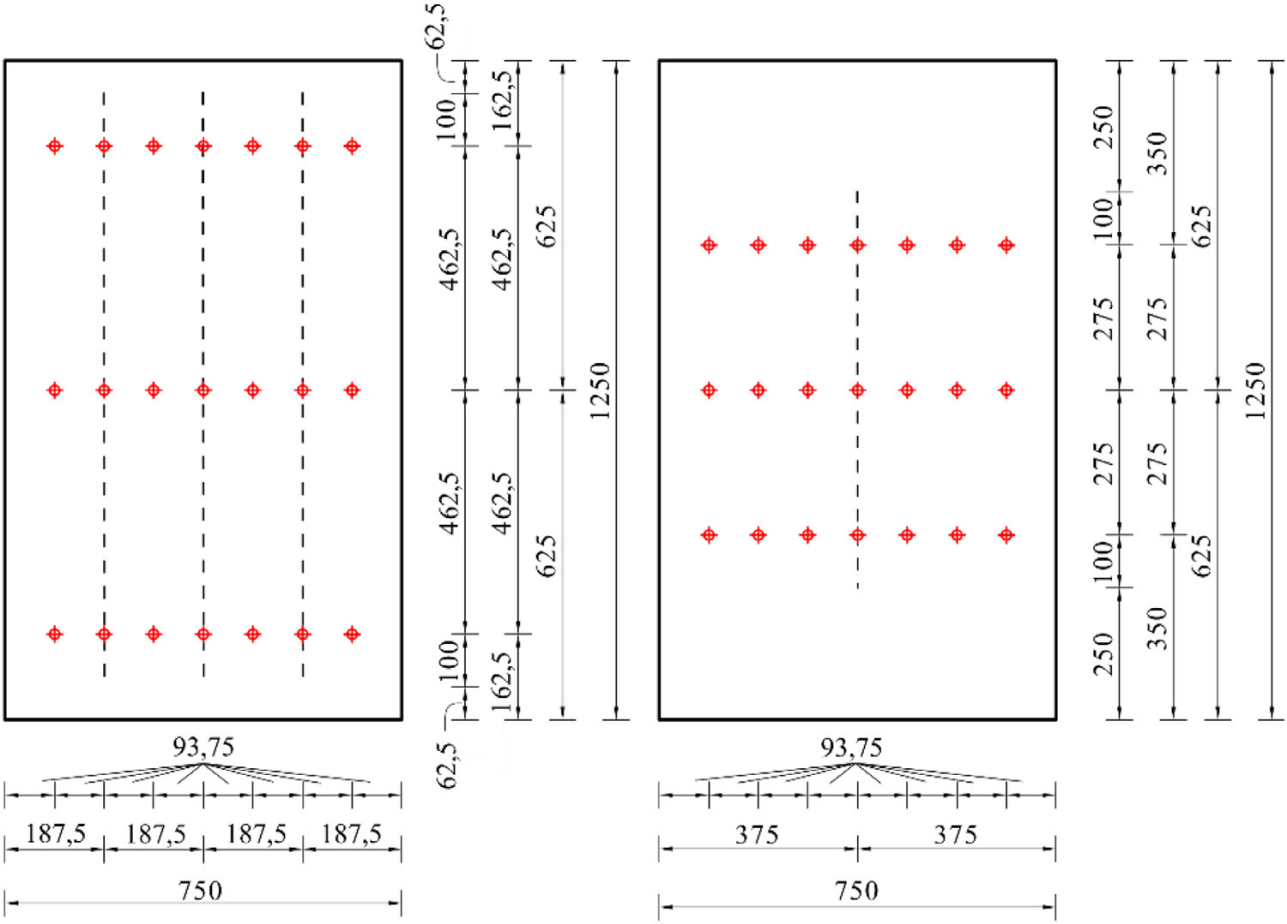
Positioning the strain gauges for the open-section specimens V_n3_g05_lss2_open (left) and V_n1_g20_lsw2_open (right). The stiffeners’ position is marked with dashed lines [1].

**Fig. 9:**
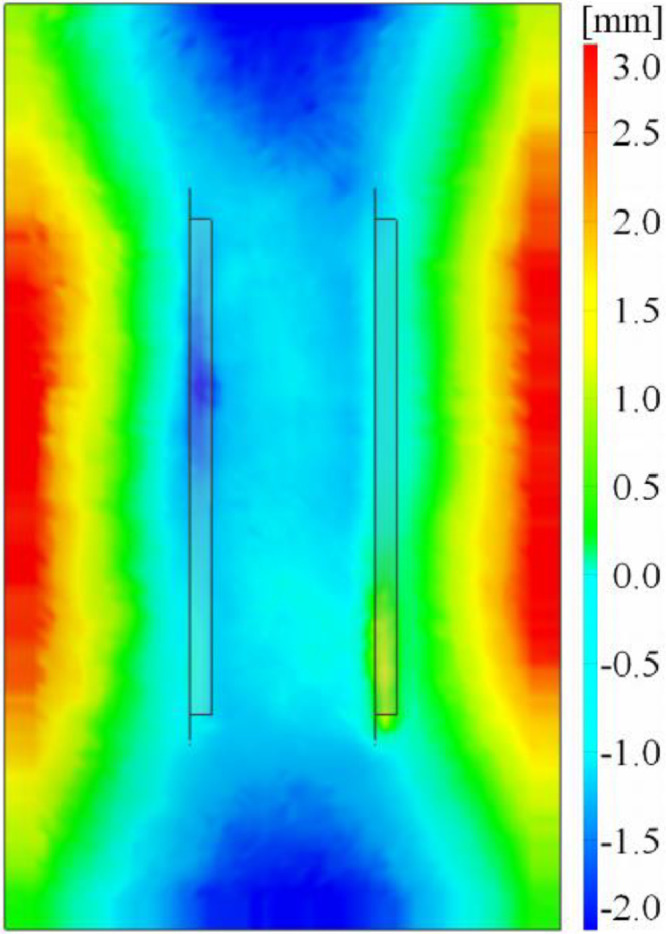
Characteristic imperfection shape on the example of specimenV_n2_g20_lsw_open [1].

Besides to tracking the specimens’ displacement field, DIC is able to measure initial imperfections. The exported point cloud needs to be cleaned of outliers and can further be applied to a finite element model, as demonstrated in Fig. 9. The underlying algorithm compares the in-plane coordinates of the very dense imperfection point cloud with the node coordinates of the FE model. It deflects the FE node based on nearest point cloud data point in the out-of-plane direction.

### 4.3 Material properties

**Fig. 10:**
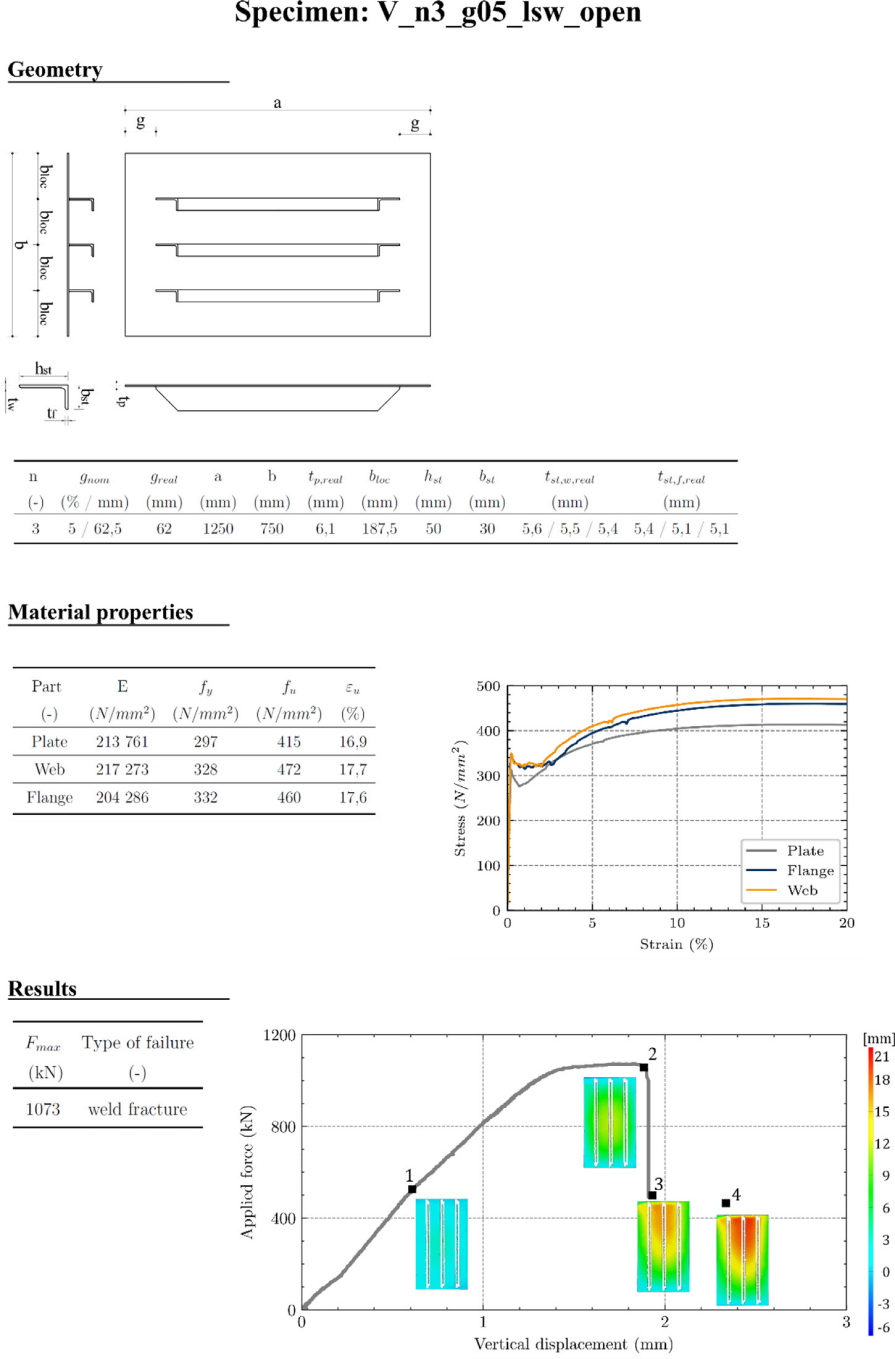
One-page results overview on the example of V_n3_g05_lsw_open.

S235JR was used to comply with the maximum hydraulic piston force. The tests have been performed in two series. In case of the open-section test series, no test certificates have been available, so that tensile coupon tests of the plates, stiffener webs and flanges were performed according to EN 6892-1 [11]. The stiffeners were tested due to suspected higher strengths in the rolled sections. Additionally, isotropy was proved by performing tensile coupon tests in three angles (0°, 45°, 90°) of the plate. In case of the closed-section test series, chemical composition was proved by test certificates. Nonetheless, control tensile coupon tests have been performed randomly in plate and stiffeners (10 times each) to verify the certificates. As demonstrated in Fig. 10, Material properties (*E, f_y_, f_u_, ε_u_*) and corresponding stress-strain-curves are specified in the one-page results overview (folder 01_overview_results_specimens) for every specimen.

## Limitations

There is no DIC-measurement of specimen *V_n3_g05_lss1_open.*

## Ethics Statement

The authors have read the ethical requirements for publication in Data in Brief and confirm that the current work does not involve human subjects, animal experiments, or any data collected from social media platforms.

## CRediT authorship contribution statement

**Immo Lukas:** Writing – original draft, Visualization, Project administration, Methodology, Investigation, Funding acquisition, Formal analysis, Conceptualization. **Ralph Timmers:** Writing – review & editing, Supervision, Project administration, Methodology. **Melanie Ropele:** Investigation, Formal analysis. **Robert Lang:** Writing – review & editing, Supervision.

## Data Availability

rearchdata UIBK.Experimental data of structural tests on non-continuously stiffened steel panels subjected to uniform compression (Original data)
